# Bibliometric and visualization analysis of risk management in the doctor–patient relationship: A systematic quantitative literature review

**DOI:** 10.1097/MD.0000000000037807

**Published:** 2024-04-19

**Authors:** Hui Li, Chenchen Zhang, Limin Li, Tong Liu, Liping Zhang, Jiqing Hao, Jiangjie Sun

**Affiliations:** a Health Management College, Anhui Medical University, Hefei, China; b First Clinical Medical College, Anhui Medical University, Hefei, China; c School of Marxism, Anhui Medical University, Hefei, China; d Clinical Medical College, Anhui Medical University, Hefei, China; e School of Management, Hefei University of Technology, Hefei, China.

**Keywords:** bibliometric analysis, CiteSpace, doctor–patient relationship, risk management, systematic quantitative literature review, VOSviewer

## Abstract

**Objectives:**

This paper analyzed the research on risk management in the doctor–patient relationship (DPR) based on a systematic quantitative literature review approach using bibliometric software. It aims to uncover potential information about current research and predict future research hotspots and trends.

**Methods:**

We conducted a comprehensive search for relevant publications in the Scopus database and the Web of Science Core Collection database from January 1, 2000 to December 31, 2023. We analyzed the data using CiteSpace 6.2.R2 and VOSviewer 1.6.19 software to examine the annual number of publications, countries/regions, journals, citations, authors, and keywords in the field.

**Results:**

A total of 553 articles and reviews that met the criteria were included in this study. There is an overall upward trend in the number of publications issued; in terms of countries/regions, the United States and the United Kingdom are the largest contributors; *Patient Education and Counseling* is the most productive journal (17); Physician communication and patient adherence to treatment: a meta-analysis is the most cited article (1637); the field has not yet to form a stable and obvious core team; the analysis of high-frequency keywords revealed four main research directions: the causes of DPR risks, coping strategies, measurement tools, and research related to people prone to doctor–patient risk characteristics; the causes of DPR risks, coping strategies, measurement tools, and research related to people prone to doctor–patient risk characteristics; the keyword burst analysis revealed several shifts in the research hotspots for risk management in the DPR, suggesting that chronic disease management, is a future research direction for the continued development of risk management in the DPR.

**Conclusions:**

The visualization analysis of risk management literature in the DPR using CiteSpace and VOSviewer software provides insights into the current research status and highlights future research directions.

## 1. Introduction

The tension between doctors and patients is a modern issue prevalent in today’s world. It has been shown that the public in several countries is dissatisfied with the doctor–patient relationship (DPR).^[1]^ Maintaining a good DPR is crucial, and building a productive DPR is an essential part of successful healthcare.^[2]^ The DPR has existed for more than 5000 years. Today, this dynamic has transformed from a traditional therapist–patient relationship to an interaction between the care provider and the service user.^[3]^ Furthermore, as patients have greater autonomy in choosing their healthcare providers, they may switch doctors frequently, leading to increased complexity in the DPR and a higher likelihood of doctor–patient risks arising. Presently, risks to the DPR include tensions, lack of trust, ongoing medical disputes, medical violence, and other crises between doctors and patients.^[4–7]^ How to effectively manage the risks associated with the DPR and improve patient satisfaction has become a prominent research topic. It is especially crucial to control and mitigate the risks associated with the DPR. Risk management is the process of identifying, mitigating, and controlling threats to an organization.^[8]^ In healthcare, risk management is closely associated with ensuring patient safety.^[9]^ Risk management measures are widely used in clinical departments, including dentistry, obstetrics and gynecology, and dermatology,^[8,10,11]^ and are also tailored for specific diseases such as psychiatric disorders, cardiovascular diseases, cancer, and diabetes.^[12–15]^ Risk management involves the development of proactive interventions as well as postrisk control measures. At present, the research on risk management in the DPR mainly includes the causes of DPR tension, doctor–patient communication, and medical dispute prevention and resolution strategies.

A study conducted by Ferguson et al^[16]^ discovered that race, ethnicity, and language significantly influenced the quality of the DPR, particularly for patients who are not proficient in English, making it less likely for them to establish rapport with their physicians. Afterward, a study by Mustafa et al^[17]^ additionally identified the adverse effects of language barriers on the DPR. They suggested that the impact of language barriers on the DPR could be reduced by assisting medical educators and policymakers in curriculum design.^[17]^ Hamid et al^[18]^ identified a lack of time allocation to patients, poor prescription interpretation, and differentiation of patients based on their social status as major factors contributing to poor relationships with physicians from the patient’s perspective. While Kumar et al^[19]^ found that the causes of current doctor–patient tensions from the perspective of physicians include stressful work for physicians, lack of communication among physicians, incidents of malpractice among physicians, poor infrastructure of medical institutions, and the negative role of the media.

It has been shown in the past that effective and efficient communication is part of a strategy to ensure that physicians provide high-quality care to their patients.^[20,21]^ A systematic review of the impact of doctor–patient communication on health outcomes included 17 randomized controlled trials, 17 controlled studies, and 8 qualitative studies that met the criteria for studies showing that communication (skills) can achieve improved and enhanced effects on treatment-related mood and behavior.^[22]^ A study by Parker et al^[23]^ discovered that effective doctor–patient communication assists physicians in collaborating with patients to achieve improved and safer medication utilization. By exploring the relationship between communication skills, health service quality, and patient trust in primary health services, Gu et al^[24]^ showed that physicians’ communication skills influence patients’ trust through service quality. Additionally, a growing body of literature is emerging which describes the use of artificial intelligence and virtual reality to facilitate doctor–patient communication regarding surgical risk. The use of these technologies has the potential to enable personalized risk communication for individual patients and within specific healthcare settings.^[25]^

In recent decades, medical dispute cases have increased dramatically worldwide,^[26]^ and reasonable resolution of medical disputes is of great significance for easing the DPR and building a harmonious doctor–patient environment. A study by Zeng et al^[27]^ suggested the following: the emphasis should be on building hospital management systems, strengthening hospital regulatory systems, improving treatment process systems and rating systems, focusing on patient feedback on hospital quality and outcomes, and combating medical violence as ways to resolve doctor–patient disputes. Meanwhile, a study by Hamid et al^[18]^ pointed out that orienting physicians to nontherapeutic care (i.e., respectful behavior, privacy, dignity, prompt attention, and clear communication) at all levels of medical education and training and improving hospital working conditions can also help prevent doctor–patient risks.

Overall, current scholars have produced fruitful results in this area. However so far, no systematic quantitative literature review (SQLR) has been conducted to assess research on risk management in the DPR. Therefore, we conducted a comprehensive bibliometric analysis of research on risk management in the DPR over the past 20 years based on a SQLR approach and using the bibliometric software CiteSpace and VOSviewer software. As far as we know, this is the first study that applies bibliometric visualization tools and combines systematic quantitative literature analysis techniques to investigate the total number of publications, countries, journals, the total number of citations, active authors, and keywords in the subject study, and then analyzed the research hotspots and trends on risk management in the DPR. We believe that this study holds significant theoretical value in revealing potential information on doctor–patient risk management and in indicating future research directions in this field.

## 2. Materials and methods

### 2.1. Data sources and collection procedures

We conducted a comprehensive search of the topic and reviewed relevant literature using the SQLR technique, searching from 2000 to 2023 (retrieved date: January 3, 2024). The data for this study were obtained from the literature collected from 2 major databases: Scopus and the Web of Science Core Collection (WOSCC), and the above data sources were used because of their broad coverage and large volume of data. Both databases collect detailed article-related information and have multiple bibliometric formats for organization and integration.

**Figure 1.**
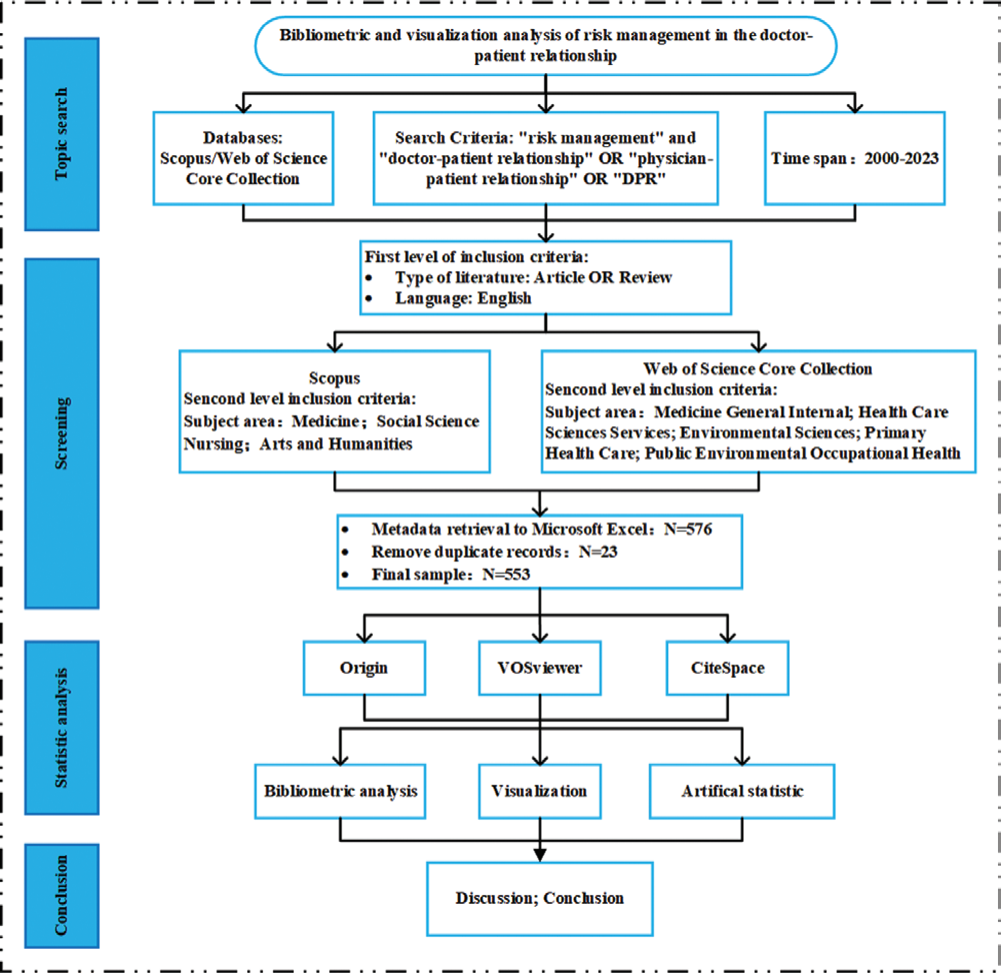
Comprehensive analytical framework for the study of risk management in the doctor–patient relationship.

First, we searched the Scopus database with the search formula ([TITLE-ABS-KEY]=(risk management OR risk governance OR risk control)) AND ([TITLE-ABS-KEY]=(doctor–patient relationship OR physician–patient relationship OR DPR)) was searched, which yielded 687 documents. According to the SQLR method, we set the scope of the literature selection to the years 2000 to 2023; the type of publication as “Article” or “Review”; the scientific results in English; in addition, we set the subject categories as the application of these exclusion criteria reduced the number of publications in the sample to 443. Second, we took the same keyword combinations to run queries on the WOSCC database, where we used exclusion criteria and restricted the results to Medicine General Internal; Health Care Sciences Services; Environmental Sciences; Primary Health Care; and Public Environmental Occupational Health, which yielded 133 documents. To avoid double counting, duplicate documents were removed across databases, resulting in a combined sample of 553 documents. We extracted relevant information about the documents included in the final sample, including title, author, total number of publications, number of citations, keywords, and country/region of affiliation. Finally, we used Origin, CiteSpace, and VOSviewer software to analyze the data, and Figure 1 depicts the scheme we followed in this study.

### 2.2. Analysis tools and methods

#### 2.2.1. Analysis tools

CiteSpace is a visual literature analysis software created by Chaomei Chen of Drexel University in the United States.^[28]^ CiteSpace software includes co-citation, co-authorship, and keyword co-occurrence,^[29]^ and its core concepts include burst detection, mediated centrality, and heterogeneous networks,^[30]^ which helps to visualize the current status of research, hot spots, and frontiers. Furthermore, a significant body of scholars worldwide have utilized it to handle mass corpora of literature to track research hotspots, research frontiers, and emerging research trends.^[31]^

VOSviewer is another bibliometric analysis software developed by van Eck and Waltman^[32]^ for constructing and viewing bibliometric maps based on web data from which key information can be extracted for a large number of publications. The multitude of features and flexibility of VOSviewer enables us to create networks based on clusters of the number of connections, coverage through temporal analysis, and density visualization using color schemes to represent occurrences rather than clusters. This deepened our understanding of relationships in the literature compared to using one form of mapping alone.^[33]^

#### 2.2.2. Bibliometric analysis and visualization

Bibliometrics is an information analysis method used to measure research trends and knowledge structure in a field and to help researchers understand current research distributions and core themes in a given area.^[34,35]^ Visualization helps to reveal the intrinsic connections between this information, such as different authors with the same research topic, research priorities of various institutions, new theories from existing institutions, etc.^[36]^ In this study, we utilized CiteSpace 6.2.R2 software and VOSviewer 1.6.19 software to perform statistical analysis on the frequency of annual publication volume, countries, journals, total citations, authors, and keywords in the field of risk management in the DPR. The aim was to clarify the distribution characteristics of publications, identify core journals, core authors, core articles, and core keywords, and construct visualization maps through keyword co-occurrence and burst analysis to reveal the evolution of topics over time and space. Additionally, we aimed to summarize existing research, uncover potential information in the field of risk management in the DPR, and explore future research hotspots and development trends. Ultimately, our goal was to provide reference directions for further research by researchers and to enhance the level and capability of risk management in the DPR. The statistical information of the statistical literature samples was also imported into Origin software to plot the corresponding graphs.

## 3. Results

### 3.1. Analysis of annual publication volume

**Figure 2.**
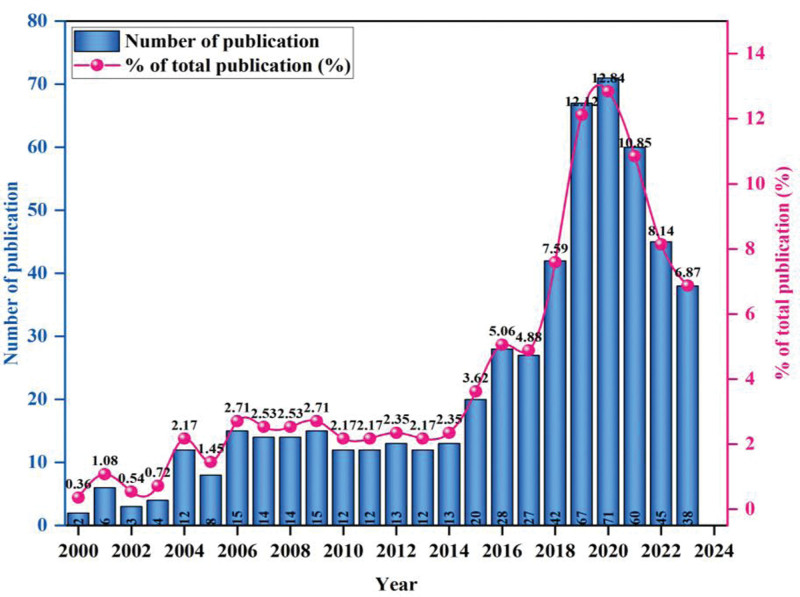
Annual trend of publications on risk management in the doctor–patient relationship (2000–2022).

The number of publications is an important indicator that reveals trends in scientific research.^[37]^ A total of 553 publications have been published on the topic of risk management in the DPR from 2000 to 2023 in the selected database. Despite some inverted S-shaped fluctuations, the overall trend in the number of publications in this subject field of research is on the rise, and the temporal distribution of research on risk management in the DPR is shown in Figure 2. The lowest output year was in 2000, with 2 papers, accounting for only 0.36%, while after nearly 2 decades of development, the highest number of papers was published in 2020, with 70 papers, accounting for 12.84%. Particularly, the number of publications in the last 5 years exceeds the total number of papers published during 2000 to 2017, reflecting the dramatic expansion of academic interest and attention to this topic.

### 3.2. Analysis of publications in different countries/regions

A total of 54 countries/regions were involved in the study of this topic. The leading countries are shown in Table 1. The top 3 countries in terms of both the number of publications and citations were the United States (214 papers, 9164 citations), the United Kingdom (53, 1927), and Australia (34, 1923). In addition, in terms of the average number of citations, the highest is the United States (41.72 citations), followed by the Australia (38.03), and United Kingdom (36.36). Among them, the United States is absolutely ahead of other countries in terms of the number of publications, total citations, and average citations, which indicated that the United States was far the most influential country in terms of the number and quality of articles in the field and has a greater contribution to the world.

**Table 1:** Leading countries in research on risk management in the doctor–patient relationship.

Country	Publications	Citations	Citations per document
United States	214	9164	42.82
United Kingdom	53	1927	36.36
Australia	34	1293	38.03
Canada	30	889	29.63
China	26	460	17.69
Germany	23	565	24.57

### 3.3. Analysis of publications of different journals

A total of 339 journals have published papers on this topic over the past 2 decades. The top 10 journals in terms of number of publications published 104 papers, accounting for 18.81%. A journal with a high number of publications means that it provides a broad platform for researchers to share their findings.^[38]^ Table 2 describes the top 10 journals that published the highest number of papers on risk management in the DPR and their specific information. It could be seen that *Patient Education and Counseling* has the highest number of papers (17), followed by the *Journal of General Internal Medicine* (14), and *BMJ Open* (13). Among them, the *Journal of Medical Internet Research* is the journal with the highest impact factor (IF; 7.4 points), and the United Kingdom is the country with a high concentration of journals.

**Table 2:** Top 10 journals in terms of the number of publications on risk management in the doctor–patient relationship.

Journal name	Publications	IF	Quartile in category	Country
*Patient Education and Counseling*	17	3.5	Q2	Ireland
*Journal of General Internal Medicine*	14	5.7	Q1	United States
*BMJ Open*	13	2.9	Q2	United Kingdom
*BMC Family Practice*	12	2.9	Q2	United Kingdom
*Family Practice*	12	2.2	Q3	United Kingdom
*BMC Health Services Research*	8	2.8	Q3	United Kingdom
*Patient Preference and Adherence*	8	2.2	Q3	United Kingdom
*Journal of Medical Internet Research*	7	7.4	Q1	Canada
*International Journal of Environmental Research and Public Health*	7	4.614	Q2	Switzerland
*Annals of Family Medicine*	6	4.4	Q2	United States

IF = impact factor.

### 3.4. Analysis of highly cited papers

The total citation frequency is a crucial indicator of the quality and importance of an article, and studying the citation frequency identifies the most influential papers in the field under study.^[39]^ The top 10 papers in terms of citations are shown in Table 3. Zolnierek K.B.H. and DiMatteo M.R.’s 2009 article in *Medical Care*, a meta-analysis on physician communication and patient adherence to treatment, had the highest number of citations at 1637, followed by Makoul G. and Clayman M.L.’s 2006 article in *Patient Education and Counseling* (1010) and Ciechanowski P.S. et al published in 2001 in the *American Journal of Psychiatry* (419), showing that the top 3 cited articles were all published before 2010 and were closely related to treatment are closely related.

**Table 3:** Top 10 papers cited on risk management in the doctor–patient relationship.

Title	Journal name	Authors	IF	Citations
Physician communication and patient adherence to treatment: a meta-analysis	*Medical Care*	Zolnierek K.B.H. and DiMatteo M.R. (2009)	3	1637
An integrative model of shared decision-making in medical encounters	*Patient Education and Counseling*	Makoul G. and Clayman M.L. (2006)	3.5	1010
The patient-provider relationship: attachment theory and adherence to treatment in diabetes	*American Journal of Psychiatry*	Ciechanowski P.S. et al (2001)	17.7	419
Racial differences in the use of BRCA1/2 testing among women with a family history of breast or ovarian cancer	*JAMA*	Armstrong K. et al (2005)	120.7	362
Switching doctors: predictors of voluntary disenrollment from a primary physician’s practice	*Journal of Family Practice*	Safran D.G. et al (2001)	0.6	244
Racial disparities-associated COVID-19 mortality among minority populations in the US	*Journal of Clinical Medicine*	Alcendor D.J. et al (2020)	3.9	203
An integrative model of shared decision-making in medical encounters	*Patient Education and Counseling*	Makoul G. and Clayman M.L. (2006)	3.5	199
Adherence with colorectal cancer screening guidelines: a review	*Preventive Medicine*	Subramanian S. et al (2004)	5.1	171
Measuring patients’ experiences with individual primary care physicians: results of a statewide demonstration project	*Journal of General Internal Medicine*	Safran D.G. et al (2006)	5.1	169
Improving care and promoting health in populations: standards of medical care in diabetes-2020	*Diabetes Care*	Amer Diabet Assoc (2020)	16.2	168

COVID-19 = coronavirus disease 2019, IF = impact factor.

### 3.5. Analysis of active author group cooperation

**Figure 3.**
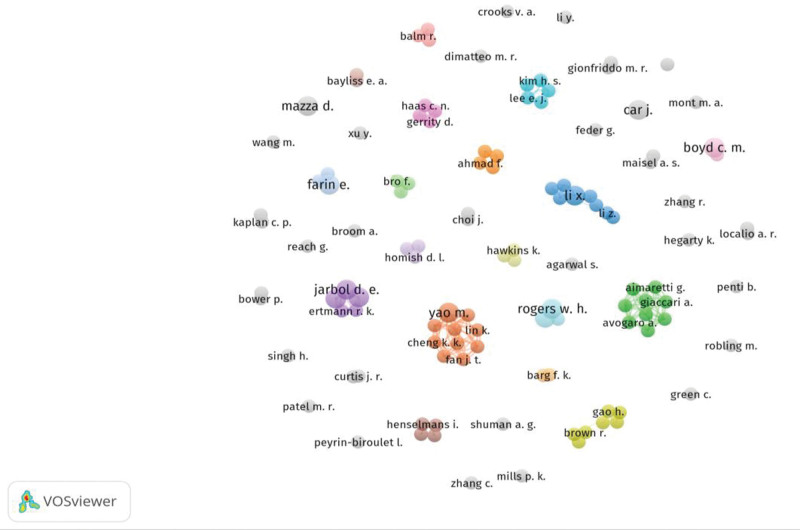
Highly cited authors and their collaborations.

A statistical analysis of the authors about the 553 papers screened above revealed a total of 2885 authors involved in research on risk management in the DPR worldwide. The 118 highly cited authors were mined, and the cited scholars and their collaborations in the field of risk management in the DPR were mapped as shown in Figure 3. Among the 513 publications, highly cited scholars such as Jarbol D.E., Sondergaard J., Pedersen L.B., Rogers W.H., Safran D.G., Boyd C.M., Car J., Farin E., Li X., Mazza D. emerged and formed a collaborative network with clusters of highly cited scholars. The top 10 authors in terms of the number of publications are shown in Table 4, and the largest number of publications is 4, which indicates that a stable and obvious core team has not yet been formed in this field.

**Table 4:** Top 10 productive authors of risk management in the doctor–patient relationship.

Rank	Authors	Publications	Institution	Country
1	Car J.	4	Nanyang Technological University	Singapore
2	Farin E.	3	University Freiburg	Germany
3	Jarbol D.E.	3	University of Southern Denmark	Denmark
4	Sondergaard J.	3	University of Southern Denmark	Denmark
5	Li X.	3	China University of Mining and Technology	China
6	Pedersen L.	3	The Arctic University of Norway	Norway
7	Pedersen L.B.	3	Tufts-New England Medical Center	United States
8	Safran D.G.	3	New England Medical Center	United States
9	Boyd C.M.	3	The Johns Hopkins University School of Medicine	United States
10	Mazza D.	3	Monash University	Australia

### 3.6. Analysis of research hotspots

**Figure 4.**
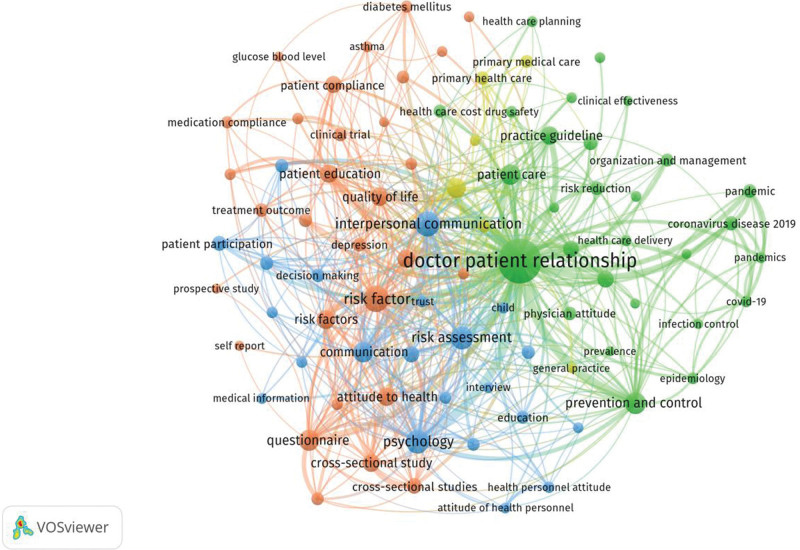
Keyword co-occurrence map on risk management in the doctor–patient relationship.

Keyword analysis can represent one of the most effective ways to understand the development of the literature over time.^[40]^ Based on VOSviewer software author keywords that appeared at least 20 times in the paper were included in the analysis and divided into 4 groups, as shown in Figure 4. Out of 4445 keywords, a total of 124 met the threshold value. The keyword “doctor patient relationship” (total link strength 2161) appeared most frequently, 252 times (11.66%), followed by “controlled study” (139 times, 11.62%), “risk factor” (101 times, 11.23%), “interpersonal communication” (96, 11.28%), “psychology” (87, 10.18%). Publications related to risk management in the DPR were divided into 4 color clusters: cluster 1 (red): deals with studies related to the causes of DPR risks, such as “communication,” “patient care,” “patient satisfaction,” “health care quality,” “covid-19”; cluster 2 (blue): keywords related to risk mitigation in the DPR, such as “patient education,” “practice guideline,” “disease control,” “patient compliance,” “quality of life”; the third group (green) involves keywords related to tools and methods for identifying risks in the DPR, such as “questionnaire,” “surveys and questionnaires,” “qualitative research,” “risk assessment”; the 4th group (yellow) relates to keywords associated with populations with characteristics that predispose them to DPR risk, such as “diabetes mellitus,” “glucose blood level,” “dependent diabetes,” “self care,” and “very elderly.

### 3.7. Trend analysis of theme evolution

**Figure 5.**
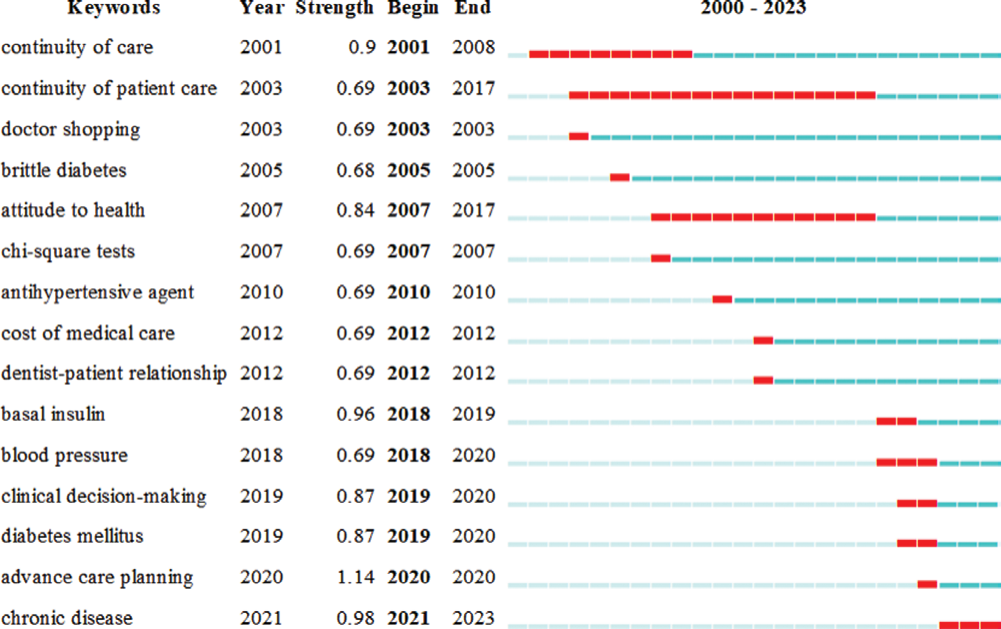
Spatio-temporal burst analysis of core keywords on risk management in the doctor–patient relationship.

The analysis of burst words reveals keywords that change rapidly or increase dramatically in number in a short period of time, allowing the emergence of research hotspots and frontiers at different times, and is a key indicator of hotspots and emerging trends in the research field.^[41,42]^ To better understand the temporal trends of the study topics, we used CiteSpace to analyze the topic burst keywords, as shown in Figure 5. The first 15 bursts of keywords were selected, and all keywords had an intensity of more than 4. The first 15 bursts of keywords were selected, the keyword with the highest intensity of occurrence was “advance care planning” (1.14) and the keyword with the longest duration was “continuity of patient care” (2003–2017). During the period 2000 to 2010, keywords such as “continuity of care,” “continuity of patient care,” “doctor shopping,” and “antihypertensive agent” were highlighted, emphasizing the importance of continuous patient care in mitigating doctor–patient risks; in the period of 2010 to 2020, the focus is on analyzing the causes of the DPR, such as medical cost, clinical decision-making, etc. The keywords that emerged in this period are “cost of medical care,” “dentist–patient relationship,” and “clinical decision-making”; With the global outbreak of Coronavirus disease 2019 (COVID-19) at the end of 2019, the keyword “chronic disease” has been popping up in recent years, suggesting that focusing on the treatment of chronic diseases has become an important aspect of managing the DPR in the wake of the COVID-19 outbreak.

## 4. Discussion

In this study, we conducted a comprehensive bibliometric analysis of the field of DPR risk management. A total of 553 scientific publications were retrieved from the Scopus database and the WOSCC database, and the development of DPR risk management over the past 20 years was analyzed. This paper systematically summarizes the development and trends of the research on risk management in the DPR, which is of great theoretical value for mining potential information in the field of doctor–patient risk management and indicating future research directions in this field. Our study identified the most productive countries, journals, and active authors. In addition, trends in the evolution of research themes in the risk management of the DPR were identified, and the most important research themes in the risk management of the DPR.

The study revealed a general increase in the number of publications focusing on DPR risk management. Notably, there has been a rapid rise in the number of publications over the past 5 years, with the highest number reached in 2020. This trend indicates that DPR risk management continues to be a significant area for future research. The sudden increase in global scholarly interest in a particular area is bound to be a common problem.^[41]^ For example, the outbreak of COVID-19 in late 2019 to early 2020. Globally, the countries at the forefront of research on risk management in the DPR are the United States and the United Kingdom, with both fruitful research results and a relatively high total and average number of citations per article. In particular, the United States is the absolute leader in terms of volume, total citations, and average citations, and the current trend will not change for a long time to come. In terms of volume, China ranks 5th in the world in this field, but the total number of citations and the average number of citations are significantly lower than those of other leading countries in terms of volume. This means that although China is paying more attention to the field of risk management in the DPR, the quality of its articles still needs to be improved.

In terms of journals, *Patient Education and Counseling* is the most popular journal, with the highest number of publications on risk management in the DPR. The *Journal of Medical Internet Research* is the journal with the highest IF of 7.4, but does not have the most publications on this topic. The IF of a journal is not positively correlated with the number of articles published. This may be related to the period time of journal submission, difficulty, and publication time. Of the top 10 journals in terms of publications, 5 were from the United Kingdom, 2 from the United States, and the remaining 3 from Ireland, Canada, and Switzerland. This indicates that European countries, represented by the United Kingdom, have a greater interest in publications in the field of risk management in the DPR and a solid research base exists.

The number of citations refers to the number of times the paper has been cited in references by other papers since it was published, up to now. The number of paper citations is a measure of how well a country’s scientific literature is recognized by other countries or institutions. The study found that the top 10 cited papers were generally concentrated between 2001 and 2009, which indicates that the papers published during this period on risk management in the DPR are more classical and recognized by most scholars worldwide. These highly cited papers provide insight into the research directions and hot spots in the field.^[38]^ Our analysis of highly cited papers showed that they focused on 4 aspects of risk management in the DPR: how to improve the DPR to enhance patient adherence^[43–46]^; exploring the contribution of doctor–patient interaction to aspects of disease counseling,^[47]^ quality tests of doctor–patient interaction,^[48]^ and measuring patients’ perceptions of the DPR.^[49]^ Highly cited authors are important drivers of scholarly innovation and disciplinary development in a field,^[50]^ and the largest number of publications is 4, which indicates that a stable and obvious core team has not yet been formed in this field.

Keyword co-occurrence analysis can reveal research directions and hotspots in a particular discipline.^[51]^ A study of documents related to risk management in the DPR identified 4 research priorities. Risks to the DPR arise for a variety of reasons, including those at the level of the healthcare system, physicians, patients, the media, and the law. In cluster 1, in addition to the keywords that are relatively well established in previous studies: “communication,” “patient care,” and “patient satisfaction,” COVID-19-related words such as “pandemic” and “epidemiology” also appeared frequently. This indicates that the DPR has also been transformed in the special context of COVID-19. For example, a study by Zhou et al^[52]^ found that the DPR improved during COVID-19, but remained fragile. In addition, Fipps and Rainey^[53]^ intertwined empathy, compassion, and humanistic care to reaffirm the value of the important role of the DPR during a crisis. For these undesirable factors that may cause tension between doctors and patients, relevant coping strategies were proposed, which can explain the terms in the second group. The keywords in this group mainly reflect the concept of patient-centeredness, which can be used to improve compliance with treatment through management education, disease control, and improvement of quality of life, thus easing the DPR.^[54]^ The third group focuses on DPR measurement instruments, involving keywords such as “questionnaire” and “surveys and questionnaires.” Currently, established scales for DPRs include the Patient-Doctor Relationship Questionnaire, the Wake Forest (Physician) Trust Scale, the Doctor–Patient Relationship Questionnaire-10, and others.^[55–57]^ However, the current scales primarily focus on trust, loyalty, respect, communication skills, and other dimensions of the DPR measurement. Few scales have been developed from the perspective of doctor–patient risk perception. It has been found that the level of perceived risk is effective in assessing the occurrence of doctor–patient risk and is key to the developmental dynamics of doctor–patient risk events.^[58]^ Therefore, it is necessary to strengthen the exploration of related studies and how to reduce the perceived risk and improve the DPR. In the 4th group, keywords related to the characteristics of the population at risk for the DPR were included, including “diabetes mellitus” and “very elderly.” However, in addition to chronic disease patients and age factors, people with special status, mental disorders, maternity with social problems, depression, and unemployment/drug use seem to be underrepresented in the analysis.

By analyzing the temporal and spatial emergence of core keywords in the field of risk management of the DPR each year, the research hotspots and spatial and temporal evolution patterns in this field can be accurately analyzed.^[59]^ Analysis of the temporal emergence of keywords showed that early studies focused on “continuity of care” (widely mentioned from 2001 to 2008) and “continuity of patient care” (widely mentioned from 2003 to 2017), indicating that patient care has been a hot topic of research for a long time before; the second burst peak occurred in 2010 and lasted until 2020. At this stage, the reason for risk in the DPR, such as health care costs and clinical decision-making, has become a hot topic of research; then, with the outbreak of “COVID-19,” chronic disease management became a hot topic. This is the first reliable bibliometric analysis of the topic of risk management in the DPR, and the findings of this study are expected to provide new directions and ideas for future research. However, several limitations of this study need to be acknowledged. First, we only analyzed data from the core collections of Scopus and Web of Science, and not all studies from all databases were included. Second, we analyzed only English-language publications and were unable to fully incorporate perspectives from other languages. Third, our study focused mainly on bibliometric analysis, aiming to analyze the structure of the field of risk management in the DPR, which is not as in-depth as traditional literature review studies. This calls for a more comprehensive and detailed literature review in the future.

## 5. Conclusion

Based on the analysis of the literature data obtained in the field of risk management in the DPR over the past 2 decades, this study is based on a SQLR and used bibliometric and visualization methods to uncover potential information and future research hotspots and trends in the field of risk management in the DPR. The following conclusions were obtained: the number of publications in the field of risk management in the DPR is generally on the rise and in a high development stage; the countries at the forefront of research on the management of risk in the DPR are the United States and the United Kingdom; a total of 339 journals have published papers on this topic, with *Patient Education and Counseling* having the highest number of articles (17) and the *Journal of Medical Internet Research* having the highest IF (7.4); found that Zolnierek K.B. and DiMatteo M.R. had the highest number of citations in a meta-analysis of physician communication and patient adherence to treatment, published in Medical Care in 2009, with 1637; the field has not yet to form a stable and obvious core team; Through the analysis of high-frequency keywords, it is found that the current research is mainly conducted around the following 4 directions: Keywords related to the causes of DPR risk: studies on “communication,” “patient care,” “patient satisfaction,” “quality of health care,” etc; Studies related to the mitigation of patient-physician relationship risk, such as “patient education,” “disease control,” “patient compliance,” etc; Studies related to tools and methods for identifying risks in the DPR, such as “questionnaires,” “surveys and questionnaires,” and “qualitative studies; There are also studies of people with characteristics that make them susceptible to the risk of DPRs: “diabetes,” “dependent diabetes,” “very old,” and most of these studies aim to reveal the risk factors that make them susceptible to DPRs and to prevent and control the risks of DPRs. According to the keyword spatio-temporal burst analysis chart, it can be seen that the research hotspots on the risk management in the DPR have undergone several shifts, and in recent years, under the influence of COVID-19, the DPR is bound to face new risks and challenges. Therefore, strengthening the management of chronic diseases in the future is of great significance for the rational control of doctor–patient risks.

## Author contributions

Conceptualization: Hui Li, Jiangjie Sun.

Data curation: Hui Li, Min Li Li, Tong Liu, Chenchen Zhang.

Formal analysis: Hui Li.

Funding acquisition: Liping Zhang, Jiangjie Sun.

Methodology: Hui Li, Min Li Li, Tong Liu.

Supervision: Liping Zhang, Jiangjie Sun.

Visualization: Hui Li.

Writing – original draft: Hui Li, Min Li Li, Tong Liu, Liping Zhang, Jiangjie Sun.

Writing – review & editing: Hui Li, Min Li Li, Chenchen Zhang, Tong Liu, Liping Zhang, Jiqing Hao, Jiangjie Sun.
